# Inhibition of IP3R/Ca2+ Dysregulation Protects Mice From Ventilator-Induced Lung Injury *via* Endoplasmic Reticulum and Mitochondrial Pathways

**DOI:** 10.3389/fimmu.2021.729094

**Published:** 2021-09-15

**Authors:** Liu Ye, Qi Zeng, Maoyao Ling, Riliang Ma, Haishao Chen, Fei Lin, Zhao Li, Linghui Pan

**Affiliations:** ^1^Department of Anesthesiology, Guangxi Medical University Cancer Hospital, Nanning, China; ^2^Key Laboratory for Basic Science and Prevention of Perioperative Organ Disfunction, Guangxi Medical University Cancer Hospital, Nanning, China

**Keywords:** ventilator-induced lung injury, calcium, inositol 1,4,5-trisphosphate receptor, endoplasmic reticulum stress, mitochondrial dysfunction

## Abstract

**Rationale:**

Disruption of intracellular calcium (Ca2+) homeostasis is implicated in inflammatory responses. Here we investigated endoplasmic reticulum (ER) Ca2+ efflux through the Inositol 1,4,5-trisphosphate receptor (IP3R) as a potential mechanism of inflammatory pathophysiology in a ventilator-induced lung injury (VILI) mouse model.

**Methods:**

C57BL/6 mice were exposed to mechanical ventilation using high tidal volume (HTV). Mice were pretreated with the IP3R agonist carbachol, IP3R inhibitor 2-aminoethoxydiphenyl borate (2-APB) or the Ca2+ chelator BAPTA-AM. Lung tissues and bronchoalveolar lavage fluid (BALF) were collected to measure Ca2+ concentrations, inflammatory responses and mRNA/protein expression associated with ER stress, NLRP3 inflammasome activation and inflammation. Analyses were conducted in concert with cultured murine lung cell lines.

**Results:**

Lungs from mice subjected to HTV displayed upregulated IP3R expression in ER and mitochondrial-associated-membranes (MAMs), with enhanced formation of MAMs. Moreover, HTV disrupted Ca2+ homeostasis, with increased flux from the ER to the cytoplasm and mitochondria. Administration of carbachol aggravated HTV-induced lung injury and inflammation while pretreatment with 2-APB or BAPTA-AM largely prevented these effects. HTV activated the IRE1α and PERK arms of the ER stress signaling response and induced mitochondrial dysfunction-NLRP3 inflammasome activation in an IP3R-dependent manner. Similarly, disruption of IP3R/Ca2+ in MLE12 and RAW264.7 cells using carbachol lead to inflammatory responses, and stimulated ER stress and mitochondrial dysfunction.

**Conclusion:**

Increase in IP3R-mediated Ca2+ release is involved in the inflammatory pathophysiology of VILI *via* ER stress and mitochondrial dysfunction. Antagonizing IP3R/Ca2+ and/or maintaining Ca2+ homeostasis in lung tissue represents a prospective treatment approach for VILI.

## Introduction

Mechanical ventilation (MV) is the most important life support therapy for acute respiratory distress syndrome (ARDS) (1, 2). With the prevalence of COVID-19, the number of patients using ventilator for mechanical ventilation has increased dramatically worldwide (3, 4). It is noteworthy that MV, while saving lives, may also produce or aggravate lung injury, that is, ventilator-induced lung injury (VILI) (5), which inevitably increases the morbidity and mortality of patients receiving ventilator therapy (5–7).

Biotrauma (inflammation) is one of the predominant mechanisms of VILI (8–10). Specifically, during mechanical ventilation, alveolar cells sense the excessive stretch of the lung and cause mechanical stimulation, which is converted into biochemical signals to activate the intracellular inflammatory signaling pathway and eventually lead to overproduction of inflammatory cytokines. Large numbers of inflammatory mediators produced into the blood circulation, causing systemic inflammatory response syndrome (SIRS), even resulting in multiorgan dysfunction (11, 12). But how the intracellular inflammatory signaling pathway works remains unclear. Thus, there is an urgent need to deeply study and develop potential therapeutic targets for VILI.

Endoplasmic reticulum (ER) stress together with mitochondria dysfunction have been suggested to play key roles in the development of inflammation (13–15). Various intracellular and extracellular stimuli induce ER stress, leading to the accumulation of misfolded or unfolded proteins in the ER lumen. This activates the unfolded protein response (UPR) to help maintain protein homeostasis and recover physiological function. Nevertheless, when the stress stimulus is severe or prolonged, cells are triggered to activate inflammatory and apoptotic signaling pathways (16, 17). Very recently, we reported that ER stress is involved in VILI by modulating the expression of inflammatory factors through the IRE1α/TRAF2/NF-κB signaling pathway (8). Mitochondrial dysfunction also plays a vital role in the pathogenesis of VILI which is characterized by mitochondrial depolarization, shut-down of adenosine triphosphate (ATP) production, and increased generation of reactive oxygen species (ROS) (12, 18). Therefore, the impacts of ER and mitochondrial dysfunction on VILI have been viewed and studied independently. However, this approach critically ignores the point that these organelles interact with each other through calcium (Ca2+) flux (19, 20).

Intracellular Ca2+ has been characterized as an essential signaling molecule responsible for controlling many biological processes such as excitability, exocytosis, motility, apoptosis, and transcription (21). Any disturbance in Ca2+ homeostasis can lead to a variety of pathologies (22–24). Inositol 1,4,5-trisphosphate receptor (IP3R) is the most ubiquitous intracellular Ca2+ channel not only abundantly expressed in the ER but also found in the mitochondrial-associated-membranes (MAMs), the close physical and functional contacts between the ER and mitochondria (25). Activation of IP3R in response to cell stimulation can result in unchecked Ca2+ release from the ER into the cytosol as well as the mitochondria (26), leading to disrupted Ca2+ homeostasis, with accompanying ER stress and mitochondrial dysfunction (27–29). Thus, the known functions of IP3R/Ca2+ suggest a tantalizing link to the disruptions in ER and mitochondria observed in VILI.

The present study therefore explored whether IP3R-induced Ca2+ release was involved in the inflammatory pathophysiology of VILI *via* ER and mitochondrial pathways. To address this hypothesis, our approach involved a murine model of VILI together with validation using *in vitro* models.

## Materials and Methods

### Animals and Reagents

Adult male C57BL/6 mice (25 ± 2 g) were purchased from the Animal Center of Guangxi Medical University (Nanning, China). The animals were maintained under standard conditions of 22°C ± 2°C and a 12 h:12 h light/dark cycle with food and water supplied ad libitum. All experiments were approved by the Institutional Animal Care and Use Committee of Guangxi Medical University Cancer Hospital.

Reagents used in this study included IP3R agonist carbachol (Abmole Bioscience, USA), IP3R inhibitor 2-aminoethoxydiphenyl borate (2-APB, Tocris Biosciences, UK), and Ca2+ chelator BAPTA-AM (MedChemExpress, USA). Carbachol was delivered in ddH2O. 2-APB and BAPTA-AM were delivered in the solution containing 1%DMSO, 4%PEG300, 0.5%Tween-80 and 94.5% ddH2O. The mice were injected subcutaneously with 1 mg/kg of carbachol 0.5 h before MV. Approximately 300μg/day of 2-APB were intraperitoneally injected for 7 days prior to MV. 1.25, 2.5 and 5 mg/kg BAPTA-AM were respectively intraperitoneally injected 0.5 h before MV. Animals’ behaviours were normal after dosing with drugs.

### MV Model and Sample Collection

The animal model was implemented based on previous study (8). Briefly, 100 mg/kg ketamine (Humanwell, China) and 10 mg/kg xylazine (Humanwell, China) were used to anesthetize mice, and a quarter of the initial dose of ketamine was added to the mice at 50 min intervals. Animals were orotracheally intubated with a 20-gauge sterilized plastic catheter (Cusabio, China), then connected to an animal ventilator (Kent Scientific Corporation, USA). The ventilation rate was 80 breaths/min, and the fraction of inspired oxygen was approximately 40–50%. The inspiration-to-expiration ratio was maintained at 1:1, and no positive end expiratory pressure was included. Mice with MV were ventilated at 20 mL/kg (high tidal ventilation, HTV) for 4 h, whereas control mice underwent orotracheal intubation but breathed spontaneously. During ventilation, vital signs of mice were indirectly assessed by looking at the mucous membranes of the mouths and the skin color of the limbs, by feeling the temperature of the limbs, and by counting the heart rates to ensure that animals were alive. After MV or spontaneous breathing, all mice were sacrificed by using a lethal dose of anesthetic agent. The bronchoalveolar lavage fluid (BALF) and lung tissue were collected and stored at −80°C, except the right lung, which was used for Hematoxylin-Eosin (H&E) staining, wet/dry (W/D) ratio calculation and transmission electron microscopy (TEM) examination. For single-cell suspensions preparation, lung tissues should be treated immediately to make sure they are fresh. It should be noted that all animal procedures were performed with great care and the whole process needs to be sterile to minimize the activation of inflammation.

### Histopathology

To assess morphological changes in lung tissues, the right lower lung lobe was fixed in 4% formaldehyde, embedded with paraffin, sectioned (4 μm) and mounted on Superfrost Plus microscope slides (Thermo, USA). After deparaffinization, the sections were stained with H&E. The degree of lung injury measured as acute lung injury scores was estimated using standard protocols as reported previously (30).

### TEM

Mice were perfused with saline and then with fixative buffer containing: 2.5% glutaraldehyde, 2.5% paraformaldehyde in 0.1 M sodium cacodylate buffer (pH 7.4). 1-2 mm cubes of lung tissues incubated in the same buffer with 1% osmium tetroxide for 1h and then immersed in 2% uranyl acetate for 2h, dehydrated in graded alcohols and propylene oxide. Ultrathin sections were stained with uranyl acetate and lead citrate and observed using a HT7800 transmission electron microscope (Hitachi, Japan). TEM analysis was performed double blinded using ImageJ (National Institutes of Health, USA). The ER and mitochondrial contacts were quantified as reported previously (31). The mitochondrial and ER membranes were delineated using the freehand tool. The selected areas were converted to masks and perimeter of ER were calculated. For the acquisition of MAM quantification, we normalized the total ER connected to mitochondria to total ER perimeter. The analysis of MAM width was performed at a 40,000×amplification.

### W/D Weight Ratio of Lung Tissue

Lung W/D weight ratio was performed as a parameter of pulmonary edema formation. The middle lobe of the right lung was removed, the moisture of the lung surface was gently absorbed using gauze, then the lungs were weighed (wet weight). The lungs were dried to a constant weight at 60°C for 48 h, and then weighed again for the dry weight.

### Inflammation Severity in the Lungs

Protein level in the BALF supernatant was measured for pulmonary permeability using a bicinchonininc acid (BCA) assay (Pierce, USA), and cells were counted by hemocytometer to determine inflammatory infiltration. Moreover, the concentrations of interleukin-1 beta (IL-1β), interleukin-6 (IL-6) and tumor necrosis factor-alpha (TNF-α) in the BALF were detected by ELISA kits (Cusabio, China) according to the manufacturer’s protocols.

### Isolation of ER and MAM Fractions

ER and MAM were isolated from lungs based on published protocols (32). Briefly, samples were washed and grinded in a stainless-steel high-speed tissue grinder (Servicebio, China). The homogenate was transferred to a 30 mL polypropylene centrifugation tube and centrifuged at 740g for 5 min at 4°C, then the supernatant was collected and the pellet (containing unbroken cells and nuclei) was discarded. The remaining supernatant was centrifuged again at 740g for 5 min at 4°C. Collect the supernatant, discard the pellet (if present) and centrifuge at 9,000*g* for 10 min at 4°C. Store the supernatant (this is a cytosolic fraction containing lysosomes and microsomes) at 4°C up to 1.5 h to proceed with further separation of ER fractions (100,000g for 1 h). Then gently resuspend the pellet containing mitochondria in 20 ml of ice-cold IB-2: 225-mM mannitol, 75-mM sucrose, 0.5% BSA and 30-mM Tris–HCl pH 7.4. Centrifuge mitochondrial suspension at 10,000*g* for 10 min at 4°C. Discard the supernatant and resuspend the crude mitochondrial pellet in 2 ml of ice-cold mitochondria resuspending buffer: 250-mM mannitol, 5-mM HEPES (pH 7.4) and 0.5-mM EGTA. Crude mitochondria were centrifuged 95,000g for 30 min on the top of a Percoll gradient (15–30%) to obtain MAMs (interphase). Then, MAMs were collected following centrifugation at 100,000g for 1 hour.

### Single-Cell Suspensions

Lung tissues were harvested to prepare monoplast suspension (33). Briefly, the whitening lungs were minced by Mayo-Noble scissors and digested with 5000 U/mL collagenase type IV, 20 U/mL DNase, and 5% fetal calf serum, then incubated at 37°C for 40 min. Cells were filtered using a 100 μm cell strainer to obtain single-cell suspensions for the subsequent Ca2+ imaging and ROS measurements.

### Cell Culture

MLE12 cells (mouse lung epithelial cells) were maintained in Dulbecco’s modified Eagle’s medium (DMEM) supplemented with 10% fetal bovine serum (FBS) and 1% penicillin/streptomycin (Invitrogen, USA). RAW 264.7 cells (mouse macrophages) were maintained in complete medium consisting of DMEM with 4.5 g/L glucose containing 10% FBS and 1% penicillin/streptomycin. All cells were incubated in a humidified atmosphere with 5% CO2 at 37°C.

### Cell Proliferation Assay

Cell proliferation was determined using the CCK-8 assay (Beyotime, China). Briefly, MLE12 and RAW 264.7 cells were plated in 96-well plates at the concentration of 5 × 103 cells/well for 24h before treatment with carbachol (0, 10, 50 and 100μM) for a further 24 h. The medium was removed and the cells washed with 1× PBS before the addition of 10 μL CCK-8 solution/well and incubation for 1 h at 37°C. Thereafter, the absorbance at 450 nm was measured using a spectrophotometer (Shimadzu, Japan).

### Apoptosis Assays

Apoptosis was measured by using the annexin V-fluorescein isothiocyanate/propidium iodide (V-FITC/PI) apoptosis detection kit (BD Pharmingen, USA) according to the manufacturer’s instructions. Briefly, after the indicated carbachol treatments, MLE12 and RAW 264.7 cells were harvested and stained with annexin V-FITC/PI at 37°C in the dark before analyzing the samples by flow cytometry (BD Biosciences, USA).

### Ca2+ Imaging

Specific fluorescent probes, Fluo-4 AM (Beyotime, China), Rhod-2 (Genmed Scientifics Inc., USA) and Mag-Fluo-AM (Genmed Scientifics Inc., USA) were used to measure the cytoplasmic Ca2+ concentrations ([Ca2+]cyto), mitochondrial Ca2+ concentrations ([Ca2+]mito), and ER Ca2+ concentrations ([Ca2+]ER), respectively. Single-cell suspensions from lung tissues were preloaded with Fluo-4 AM (5 mM) by incubation at 37°C for 20 min and thereafter washed three times with PBS to remove extracellular Fluo-4 AM. Fluorescence intensity was recorded using a fluorescence microscopy (Leica, Germany) where calcium bound Fluo-4 AM emits green fluorescence when excited at 488 nm. Cells were preloaded with both 100μL of Rhod-2 and Mag-Fluo-AM diluted dyeing solutions for 30 min at 37°C in the CO_2_ incubator. The dyeing solution was then removed and 200 μL of cleaning fluid and 2 μL of permeated fluid were added, followed by incubation at 37°C for 4 min. after washed with the cleaning fluid, cells were simultaneously measure [Ca2+]mito and [Ca2+]ER using 490 nm and 550 nm excitation wavelengths, respectively.

### ROS Measurements

Intracellular ROS levels were measured using DCFH-DA (Beyotime, China) by incubating cells with 10 μM DCFH-DA at 37°C for 30 min, washing twice with PBS and subsequently analyzing by flow cytometry.

### JC-1 Staining and ATP Assays

Mitochondrial membrane potential (Δψm) was assessed using JC-1 staining (MedChemExpress, USA) according to the manufacturer’s protocols using fluorescence microscope. Images were collected at 535 nm/590 nm and 485 nm/535 nm (ex/em), respectively, and the results expressed as the ratio of total fluorescence (JC-1 aggregates: monomer). Alternatively, ATP levels were determined *via* firefly luciferase-associated chemiluminescence (Sigma-Aldrich, Germany), following the manufacturers’ instructions.

### RNA Isolation and Real-Time PCR

Total RNA was extracted from lungs using TRIzol reagent (Invitrogen, USA) according to the manufacturer’s instructions. RNA concentration/purity was determined by 260/280nm absorbance ratio, and 2 μg of total RNA converted to cDNA using the PrimeScript RT master kit (Takara, Japan). Real-time PCR reactions were performed using SYBR Green (Takara, Japan) with specific primers for GRP78, CHOP, NLRP3, Caspase-1, ASC and GAPDH. The results were normalized to GAPDH levels using the 2^−△△Ct^ method of quantification. The primer sequences are shown in **Table 1**.

**Table 1:** Sequences of the primers used to quantitate gene expression.

Genes	Primer sequences (5’-3’)
Mouse-GRP78	Forward GAAAGGATGGTTAATGATGCTGAG
	Reverse GTCTTCAATGTCCGCATCCTG
Mouse-CHOP	Forward CAAATGGCAGTTCAAAACCATC
	Reverse ATGTGTGCTGTGTGTGTGTTCC
Mouse-NLRP3	Forward TGTGAGAAGCAGGTTCTACTCT
	Reverse GACTGTTGAGGTCCACACTCT
Mouse-Caspase-1	Forward AGGCATGCCGTGGAGAGAAACAA
	Reverse AGCCCCTGACAGGATGTCTCCA
Mouse-ASC	Forward GACAGTACCAGGCAGTTCGT
	Reverse AGTCCTTGCAGGTCAGGTTC
Mouse-GAPDH	Forward TGTGTCCGTCGTGGATCTGA
	Reverse TTGCTGTTGAAGTCGCAGGAG

### Western Blot Analysis

Proteins were extracted from lungs with RIPA buffer (Solarbio, China) supplemented with protease inhibitors (Roche, Germany). Nuclear and cytoplasmic proteins were extracted using the Nuclear and Cytoplasmic Protein Extraction Kit (Beyotime, China). The protein concentrations were measured using BCA assay and equal protein amounts/sample electrophoresed on 10% SDS polyacrylamide gels and subsequently transferred onto polyvinylidene fluoride (PVDF) membranes (Millipore, USA). The membranes were blocked with 5% milk in TBST for 1 h at room temperature, incubated with anti-IP3R1 (A4436, ABclonal), anti-FACL-4 (sc-365230, Santa Cruz), anti-Calnexin (AP0635, ABclonal), anti-Tubulin (#2148, CST), anti-GRP78 (sc-166490, Santa Cruz; and/or GB11098, Servicebio), anti-CHOP (sc-7351, Santa Cruz), anti-phospho-IRE1α (ab48187, abcam), anti-IRE1α (ab37073, abcam), anti-TRAF2 (#4724, CST), anti-XBP-1s (#40435, CST), anti-phospho-PERK (#3179, CST), anti- PERK (#5683, CST), anti- phospho- eIF2α (AP0635, ABclonal), anti- eIF2α(A0764, ABclonal), anti-ATF6 (ab37149, abcam), anti-IκBα (#4814s, CST), anti-p-NF-κB p65 (Ser536, #3033s, CST), anti-NF-κB p65 (#8242s, CST), anti-Lamin B (sc-374015, Santa Cruz), anti- NLRP3 (#13158, CST), anti-caspase-1 (A0964, ABclonal), anti-ASC (67824S, CST), anti-β-actin (#4970, CST). The primary antibodies were diluted 1:1000 except for anti-IP3R, which was used at 1:500. All the antibodies were incubated overnight at 4°C before further incubation with HRP-conjugated goat anti-rabbit (1:2000) or anti-mouse (1:2000) secondary antibodies (Beyotime, China) and visualization of protein bands using enhanced chemiluminescence (ECL).

### Immunofluorescence

Sections from the paraffin-embedded lung tissues were dewaxed in xylene and hydrated using graded alcohol before conducting antigen retrieval (Beyotime, China). Thereafter, the tissues were blocked with 2% bovine serum albumin in PBS for 1 h and then incubated with primary antibodies for GRP78 (1:500), CHOP (1:200), IP3R1 (1:500) and anti-p-NF-κB p65 (1:500) overnight at 4°C, followed by washing with PBST three times (5 min each) and staining with fluorophore-conjugated secondary antibodies (Alexa Fluor 488 or 546 nm, Invitrogen, USA) for 2 h at room temperature in the dark. Sections were finally incubated in DAPI solution (Invitrogen, USA) for nuclear staining and after washing with PBST, immunofluorescence staining was visualized and recorded using epifluorescence microscopy.

### Statistical Analysis

Statistics were performed using the SPSS software 18.0 (IBM, USA) with quantitative data presented as the means ± standard deviation (SD) throughout. Grubbs’ test was performed to identify outlying data, and there is no outlier detected in this study. Shapiro-Wilk test was used for normality, and Student’s t-test were used for comparisons between two groups when the data were normally distributed. Otherwise, Mann-Whitney U test was conducted. Differences between multiple experimental groups were compared using one-way analysis of variance followed by the LSD method. Values of P < 0.05 were considered to indicate statistically significant differences.

## Results

### HTV Increases IP3R1 Expression and Enhances MAM Formation in the Mouse Lung

**Figure 1:**
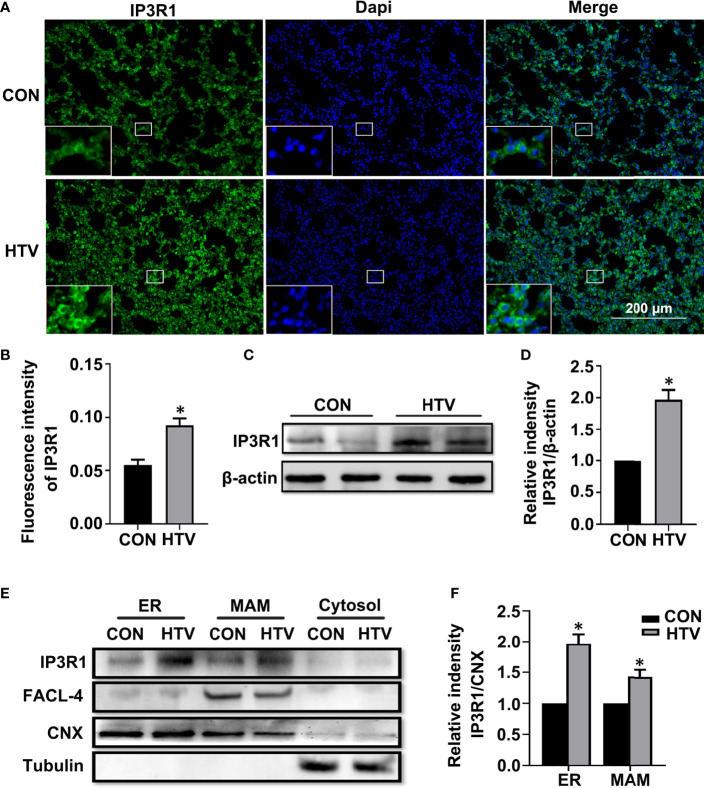
MV with HTV induces IP3R1 activation in lungs. Immunofluorescence photomicrographs **(A)** and quantification analysis **(B)** of IP3R1 in lung tissues of mice with spontaneous breathing (CON group) or mechanical ventilation at high tidal volume (HTV group). Dapi (blue) was used to stain the nuclei. Scale bar: 200μm. An inset picture was employed to show the indicated area at 4X magnification. Western blot **(C)** and quantification analysis **(D)** of IP3R1 protein expression in lung extracts. **(E)** Western blot analysis of the indicated subcellular fractions for IP3R1, calnexin (CNX; ER and MAM marker), FACL-4 (MAM marker) and tubulin (cytosolic marker). **(F)** Quantification of IP3R1 protein expression in the ER and MAM was performed by normalizing to CNX. Data are expressed as means ± SD (n = 6 animals per group). *P < 0.05 *vs*. CON group.

We first compared the IP3R levels in the lungs of control (spontaneous breathing) *versus* HTV treated mice. IP3Rs have three different isoforms. Here, we focus on IP3R1, which is the most widely expressed in tissues and is recognized as an ubiquitous type of IP3R family (34). Independent evaluation using immunofluorescence and Western blotting revealed that IP3R1 levels were significantly increased in lungs exposed to HTV (**Figures 1A–D**, respectively). To further delineate the subcellular locations associated with the increased IP3R1 levels, we performed density gradient centrifugation to derive ER and MAM-enriched fractions from mouse lung tissues. Analyses of IP3R1 in comparison to FACL-4 and CNX, markers of MAM and ER respectively, showed that there were dramatic increases in the levels of IP3R1 localizing to the MAM and ER in response to HTV (**Figures 1E, F**).

**Figure 2:**
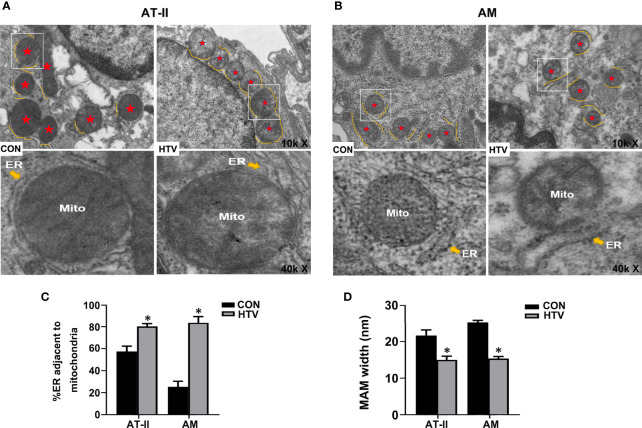
MV with HTV induces MAM formation and changes in ER and mitochondrial morphology in lung cells. Representative TEM images of lung sections derived from group CON and HTV mice at ×10,000 (top images) or ×40,000 magnifications of the boxed areas (bottom images) in **(A)** alveolar type II epithelial cells (AT-II) and **(B)** alveolar macrophage (AM). The red stars indicate mitochondria, and the yellow curve indicate the ERs which around the mitochondria (Mito). **(C)** Quantitation of ER length adjacent to mitochondria normalized to total ER length. **(D)** Quantitation of width between ER and mitochondrial. Data are expressed as means ± SD (n = 3 per group), *P < 0.05 *vs*. CON group.

Next, we used TEM to investigate whether VILI causes changes in the physical interface between the ER and mitochondria in key lung cell types. Towards this, we examined alveolar type II epithelial cells (AT-II; **Figure 2A**) and alveolar macrophage (AM; **Figure 2B**) in lung sections collected from HTV-ventilated *versus* spontaneous breathing animals. As illustrated, HTV treatment was associated with morphological rearrangement and expansion of the ER, a narrowing of the ER-mitochondrial cleft and general disruption of the mitochondria in both AT-II and AM cells. Consistently, morphometric analyses showed there were increases in the total ER membranes adjacent to mitochondria (**Figure 2C**) and well as accompanying reductions in MAM width (**Figure 2D**). Together these results demonstrate that HTV increases IP3R1 expression in MAM and ER and moreover, enhances the formation of MAM in key lung cell types.

### HTV Causes IP3R-Mediated ER Ca2+ Release in the Mouse Lung

**Figure 3:**
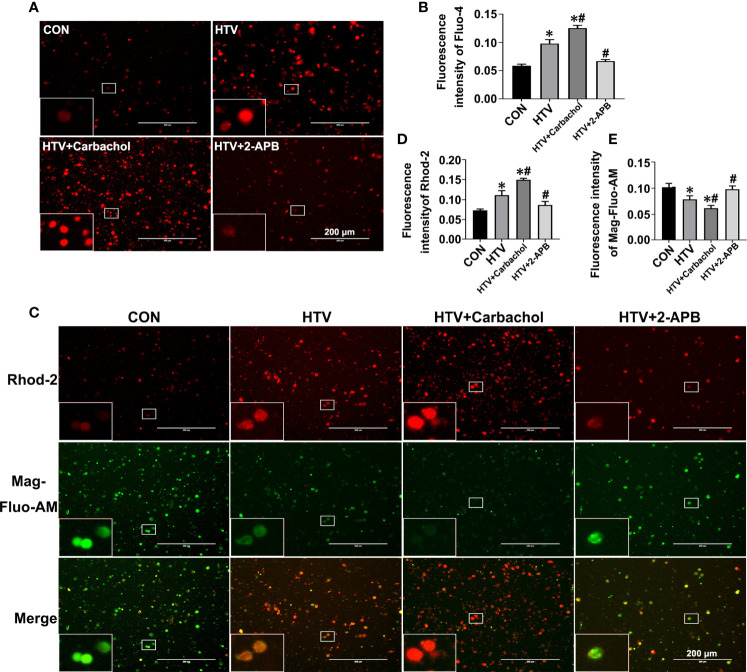
Effects of carbachol and 2-APB on HTV-induced Ca2+ homeostasis in lungs. Single-cell suspensions from lung tissues were preloaded with Fluo-4 AM, Rhod-2 and Mag-Fluo-AM, respectively. **(A)** Fluo-4 AM labeling the cytoplasmic Ca2+ in lung tissues from CON group, HTV group, IP3R agonist carbachol pretreatment upon HTV stimulation group (HTV+Carbachol group) and IP3R inhibitor 2-APB pretreatment upon HTV stimulation group (HTV+2-APB group). An inset picture was employed to show the indicated area at 4X magnification. **(B)** Fluorescence quantification analysis of Fluo-4 AM. **(C)** Rhod-2 and Mag-Fluo-AM co-dyeing to label Ca2+ in the mitochondria and ER in different groups. An inset picture was employed to show the indicated area at 4X magnification. **(D)** Fluorescence quantification analysis of Rhod-2. **(E)** Fluorescence quantification analysis of Mag-Fluo-AM. Data are expressed as means ± SD (n = 6 per group). *P < 0.05 *vs*. CON group; *^#^P* < 0.05 *vs*. HTV group.

IP3R localized to the ER/MAM functions to facilitate Ca2+ transfer into the cytoplasm and mitochondria. The observed increases in IP3R1 together with the formation of MAM in HTV mice led therefore us to postulate this treatment likely caused disruptions in Ca2+ homeostasis. To address this hypothesis, we evaluated the relative changes in intracellular Ca2+ levels using Fluo-4, Rhod-2 and Mag-Fluo-AM that measure cytoplasmic ([Ca2+]cyto), mitochondrial ([Ca2+]mito) and ER ([Ca2+]ER) Ca2+, respectively. Analyses were performed on lung tissues from nonventilated (CON) mice, HTV-only treated mice or HTV combined with pretreatment with either the IP3R agonist carbachol or the IP3R inhibitor 2-APB. Instructively, we observed that compared to the CON group, HTV facilitated significant increases in the levels of cytoplasmic Ca2+ (**Figures 3A, B**). Moreover, carbachol pretreatment further increased [Ca2+]cyto while 2-APB reversed the effects of HTV, with [Ca2+]cyto levels close to CON. Similarly, lung exposure to HTV significantly increased mitochondrial Ca2+ levels (**Figures 3C, D**) while in contrast, the ER-associated Ca2+ levels were relatively decreased by HTV (**Figures 3C, E**). Corresponding changes in both [Ca2+]ER and [Ca2+]mito in response to carbachol and 2-APB were fully consistent with IP3R-dependent ER Ca2+ release or loading, respectively. Collectively these results indicate that MV of mice with HTV promotes excessive ER Ca2+ release which is mediated through IP3R.

### IP3R/Ca2+ Signaling Is Involved in HTV-Induced Lung Injury and Inflammation

**Figure 4:**
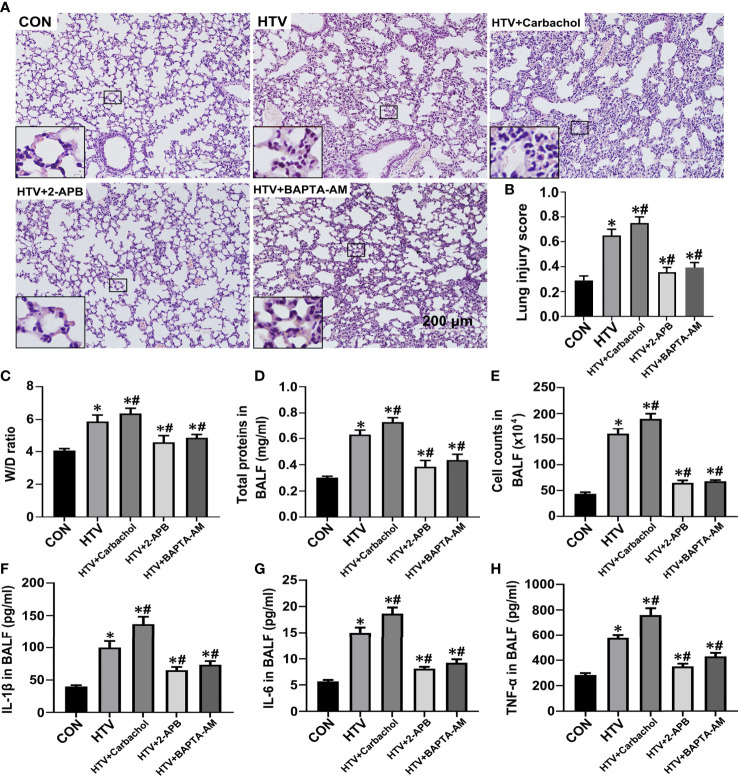
Effects of carbachol, 2-APB and BAPTA-AM on HTV-induced pathological lung injury and inflammation. **(A)** H&E staining the histology of lung tissues from group CON, HTV, HTV+Carbachol, HTV+2-APB mice, and the HTV-treated mice pretreated with Ca2+ chelator BAPTA-AM (HTV+BAPTA-AM group). Scale bar: 200μm. An inset picture was employed to show the indicated area at 4X magnification. **(B)** Pathological scores were assessed by results of H&E staining. **(C)** Lung edema was assessed by determining the weight ratio between wet and dry lungs. **(D)** Total protein concentration in BALF. **(E)** Infiltrated cell counts in BALF. **(F–H)** Levels of IL-1β **(F)**, IL-6 **(G)** and TNF-α **(H)** in BALF. Data are expressed as means ± SD (n = 8 per group except for group HTV+ Carbachol (n=7), which has 1 mouse died unexpectedly and was removed from analysis). *P < 0.05 *vs*. CON group; ^#^ *P* < 0.05 *vs*. HTV group.

To better define the role of IP3R-mediated ER Ca2+ efflux in the development of HTV-induced lung pathologies, we assessed how pretreatment with carbachol and 2-APB together with BAPTA-AM, the latter a membrane-permeable Ca2+ chelator, would affect lung injury and inflammation in VILI. Assessment of the dose-dependent effects of BAPTA-AM revealed that 2.5mg/kg was sufficient to prevent the excessive ER Ca2+ release induced by HTV (**Figure S1**). The results were assessed by histopathology, W/D ratio, BALF protein levels, the number of infiltrating cells and the levels of the inflammatory cytokines IL-1β, IL-6 and TNF-α. Similar to previous studies (8, 10), lungs from animals ventilated with HTV showed obvious lung injury including alveolar septal thickening, pulmonary edema, and inflammatory-cell infiltration (**Figure 4A**). Moreover, the lung histopathology score (**Figure 4B**), W/D ratio (**Figure 4C**), BALF protein levels (**Figure 4D**), the number of infiltrated cells (**Figure 4E**) and the levels of IL-1β, IL-6, TNF-α in BALF (**Figures 4F–H**) were notably increased in the HTV group compared with CON group. Therefore, the MV model used faithfully reproduces all of the key aspects of lung injury and inflammation known to be associated with VILI.

The application of carbachol pretreatment to HTV treated mice showed this to exacerbate morphological injury (**Figures 4A, B**) and lung edema (**Figures 4C–E**) while in contrast, HTV-induced lung edema and injury were improved by treatment with 2-APB. Consistently, the concentrations of IL-1β, IL-6 and TNF-α in BALF were significantly increased in the presence of carbachol, whereas 2-APB blocked the increase in BALF cytokine levels. Similarly, pretreatment with 2.5mg BAPTA-AM produced comparable effects to pretreating mice with 2-APB (**Figures 4A–E**), reinforcing the essential role of intracellular Ca2+ fluxes in the development of lung injury and inflammation by HTV. Altogether, these data indicate that an IP3R-Ca2+ signaling axis is involved in HTV-induced lung injury and inflammation.

### IP3R Inhibition Prevents ER Stress-Induced Inflammation in HTV-Treated Mice

**Figure 5:**
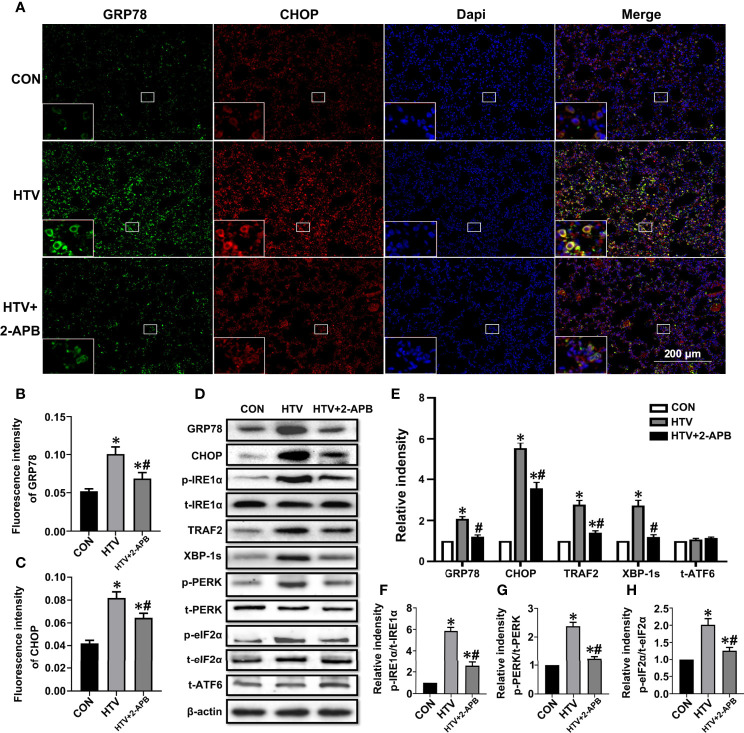
2-APB inhibits ER stress in HTV-treated mice. **(A)** Immunofluorescence photomicrographs of GRP78 (green) and CHOP (red) in lung tissues of group CON, HTV, and HTV+2-APB mice. Dapi (blue) was used to stain the nuclei. Scale bar: 200μm. An inset picture was employed to show the indicated area at 4X magnification. **(B, C)** Graphic presentations of fluorescence mean densities of GRP78 and CHOP. **(D)** Levels of GRP78, CHOP, p-IRE1α, t-IRE1α, TRAF2, XBP-1s, p-PERK, t-PERK, p-eIF2α, t-eIF2α, t-ATF6, β-actin proteins by Western blot. **(E)** Relative protein expression of GRP78, CHOP, TRAF2, XBP-1s and t-ATF6 relative to β-actin. **(F)** The relative ratio of p-IRE1α protein was presented to t-IRE1α. **(G)** The relative ratio of p-PERK protein was presented to t-PERK. **(H)** The relative ratio of p-eIF2α protein was presented to t-eIF2α. Data are expressed as means ± SD (n = 6 per group). *P < 0.05 *vs*. CON group. ^#^ *P* < 0.05 *vs*. HTV group.

We previously established that ER stress mediated through the IRE1α signaling pathway was involved in VILI. To investigate whether IP3R plays an essential role in the ER stress response seen in VILI, first, we evaluated the expression of the ER stress markers GRP78 and CHOP in lung tissues from HTV and CON mice that were pretreated with or without 2-APB. Immunofluorescence analysis of lung sections showed that the levels of GRP78 and CHOP were increased in the HTV group but their high protein levels were significantly reduced by 2-APB pretreatment (**Figures 5A–C**). Next, we comprehensively evaluated the expression of all three effector arms of ER stress (IRE1α, PERK, and ATF4), among the different groups. Western blot analysis showed that MV with HTV significantly increased the expression of GRP78, CHOP, p-IRE1α, TRAF2, XBP-1s, p-PERK and p-eIF2α, all of which were reduced by 2-APB. However, the ATF6 expression was not changed (**Figures 5D–H**), altogether indicating that IP3R affects IRE1α and PERK but not ATF6 signaling.

**Figure 6:**
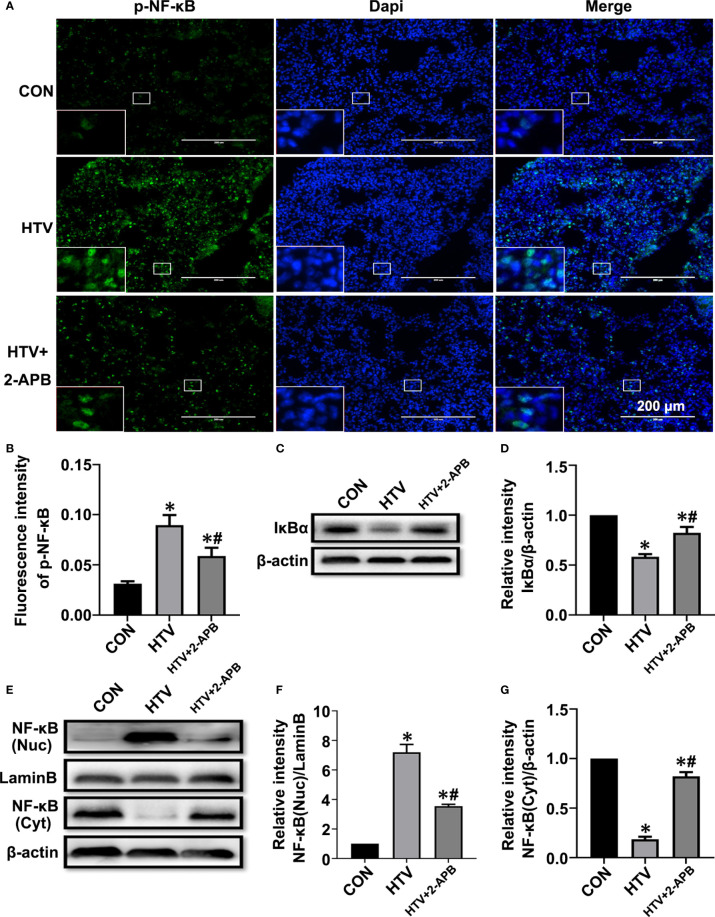
2-APB inhibits activation of NF-κB signaling pathway in HTV-treated mice. **(A, B)** Immunofluorescence photomicrographs and quantification analysis of p-NF-κB p65 in lung tissues of group CON, HTV and HTV+2-APB mice. Dapi (blue) was used to stain the nuclei. Scale bar: 200μm. An inset picture was employed to show the indicated area at 4X magnification. **(C, D)** Representative immunoblots of IκBα in lung extracts and densitometric analyses of IκBα. **(E–G)** Representative immunoblots of NF-κB p65 in nucleus and cytoplasm, and densitometric analyses of NF-κB p65. Data are expressed as means ± SD (n = 6 per group). *P < 0.05 *vs*. CON group. ^#^ *P* < 0.05 *vs*. HTV group.

Additionally, we measured NF-κB signaling responses, since the transcriptional targets of NF-κB are known to play important roles in inflammation, and moreover, NF-κB signaling is also the common inflammatory pathway invoked by ER stress. As shown in **Figures 6A, B**, the levels of activated p-NF-κB were elevated by HTV. Indeed, consistent with NF-κB pathway activation, HTV-injured lungs showed decreased levels of IκBα, the major NF-κB inhibitory protein (**Figures 6C, D**) and moreover, dramatic increases in the nuclear levels of NF-κB (**Figures 6E–G**). Instructively, the phosphorylation and translocation of NF-κB together with the degradation in IκBα, were largely blocked by 2-APB.Collectively, these results suggest that reducing IP3R activity attenuates ER stress-induced inflammation *via* IRE1α and PERK pathways in HTV-treated mice.

### IP3R Inhibition Improves Mitochondrial Dysfunction and Reverses NLRP3 Inflammasome Activation in HTV-Treated Mice

**Figure 7:**
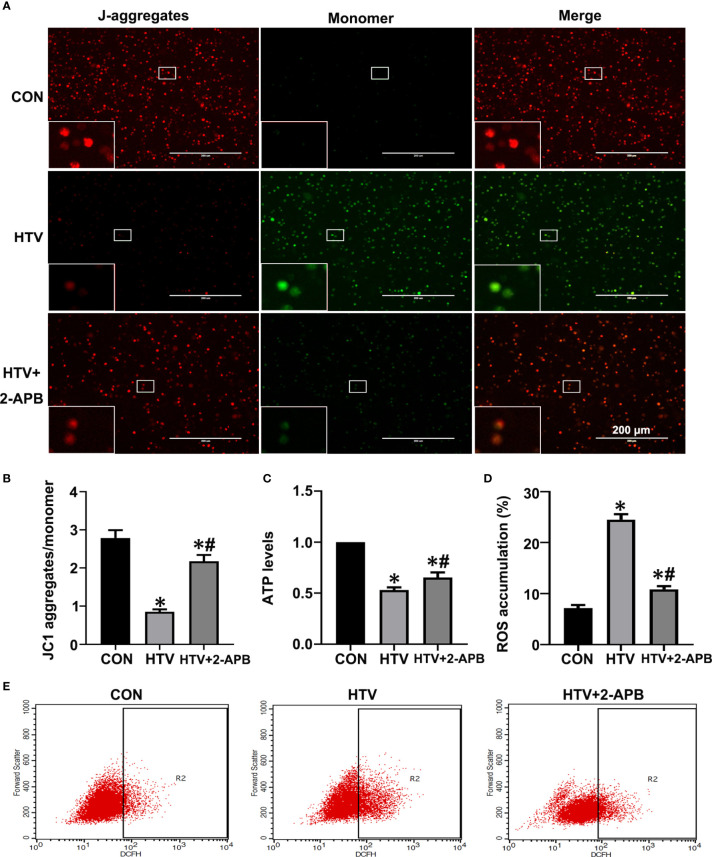
2-APB improves mitochondrial dysfunction in HTV-treated mice. **(A, B)** Fluorescence microscopy and the ratio of JC-1 staining (red, J-aggregates; green, monomer) in lung tissues from CON, HTV and HTV+2-APB group, Scale bar: 200μm. An inset picture was employed to show the indicated area at 4X magnification. **(C)** Levels of ATP. **(D, E)** Levels of ROS were determined by flow cytometry. Data are expressed as means ± SD (n = 6 per group). *P < 0.05 *vs*. CON group. ^#^ *P* < 0.05 *vs*. HTV group.

Previous studies have suggested that mitochondrial dysfunction induces activation of the NLRP3 inflammasome, which is vital for the pulmonary inflammatory response (35, 36). Given that IP3R-mediated changes in Ca2+ homeostasis affect mitochondrial Ca2+ stores as well as provoking activation of inflammatory processes, we sought to connect the effects of HTV with mitochondrial dysfunction and NLRP3 inflammasome activation. First, we measured the effects of IP3R on mitochondrial dysfunction during VILI by measuring changes in Δψm, together with ATP and ROS levels. Indeed, compared with the spontaneous breathing controls, Δψm along with ATP levels were decreased in the HTV group (**Figures 7A–C**). Alternatively, there was significantly increased ROS generation in the HTV group compared with the CON treated mice ROS (**Figures 7D, E**). Importantly, 2-APB pretreatment prevented these effects, indicative that IP3R was responsible for mitochondrial dysfunction following HTV.

**Figure 8:**
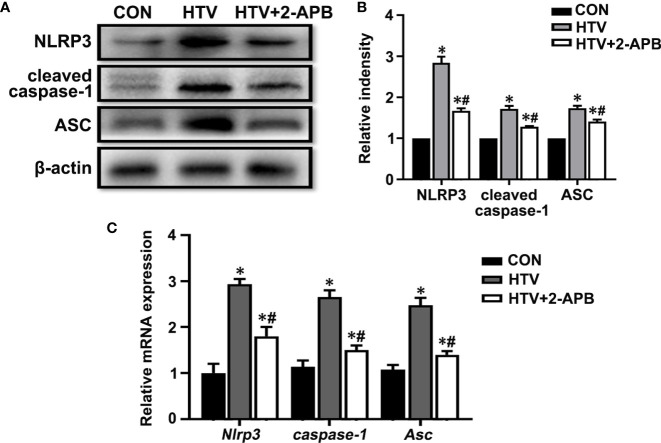
2-APB inhibits NLRP3 inflammasome activation in HTV-treated mice. **(A, B)** Representative immunoblots of NLRP3, cleaved caspase-1and Asc in lung extracts from CON, HTV and HTV+2-APB group and densitometric analyses of NLRP3, cleaved caspase-1and Asc. **(C)** Levels of NLRP3, caspase-1 and Asc mRNA. Data are expressed as means ± SD (n = 6 per group). *P < 0.05 *vs*. CON group. ^#^ *P* < 0.05 *vs*. HTV group.

Next to assess whether IP3R was also responsible for activating the NLRP3 inflammasome, we measured the levels of key mediators, namely NLRP3, caspase-1 and the ASC in lungs from the different treatment groups. Measured by both Western blot and qPCR, the results showed increases in the expression of NLRP3, caspase-1 and ASC at protein and mRNA levels in the HTV-treated group relative to the CON group (**Figures 8A–C**). Noticeably, there were increased levels of the cleaved form of caspase-1 following exposure to HTV (**Figure 8A**), indicating it activated form. Moreover, 2-APB pretreatment was able to largely suppress the increases in these mediators, indicative of the contribution of IP3R to NLRP3 inflammasome activation. Together these results suggest that controlling IP3R activity can alleviate HTV-induced mitochondrial dysfunction and prevent activation of the NLRP3 inflammasome.

### IP3R Agonist Facilitates Inflammatory Response in Lung Epithelial Cells and Macrophage

**Figure 9:**
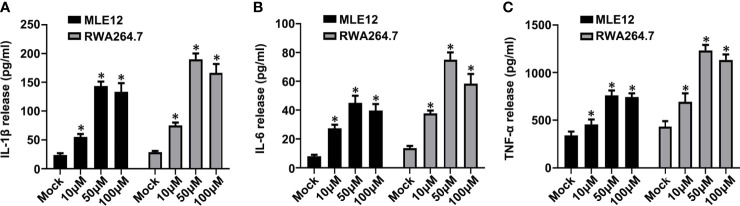
Carbachol facilitates inflammatory response in lung epithelial cells and macrophage. **(A–C)** Levels of IL-1β **(A)**, IL-6 **(B)** and TNF-α **(C)** in MLE12 and RAW264.7 cell supernatants. Data are expressed as means ± SD from 3 independent experiments. *P < 0.05 *vs*. Mock group.

To supplement the findings in our VILI mouse model, we turned to examine the effects of manipulating IP3R *in vitro* using cell lines representative of lung epithelial and resident macrophage, namely, MLE12 and RAW264.7 cells, respectively. Treating cells with carbachol (10, 50 and 100μM) showed there were dose-dependent inhibitory effects on cell viability in both MLE12 and RAW264.7 cells as measured using CCK-8 assays (**Figure S2A**). Furthermore, visual examination of the cultures revealed that 10 μM carbachol reduced cell proliferation in both lung cell lines while the 50 and 100 μM concentrations displayed cytotoxic effects (**Figure S2B**). Concurrent assessment of these cells using the annexin V- FITC/PI flow cytometric assay indicated that 50 μM carbachol efficiently induced apoptosis in both MLE12 and RAW264.7 cells (**Figure S2C**). Moreover, carbachol treatment triggered increases in the levels of IL-1β, IL-6 and TNF-α in cell supernatants, and 50 μM carbachol treatment showed the highest release of these inflammatory factors (**Figures 9A–C**). Collectively, these data that *in vitro* activation of IP3R using the agonist carbachol stimulates inflammatory responses in both lung epithelial cells and macrophage.

### IP3R Agonist Stimulates ER Stress and Mitochondrial Dysfunction in Lung Epithelial Cells and Macrophage

**Figure 10:**
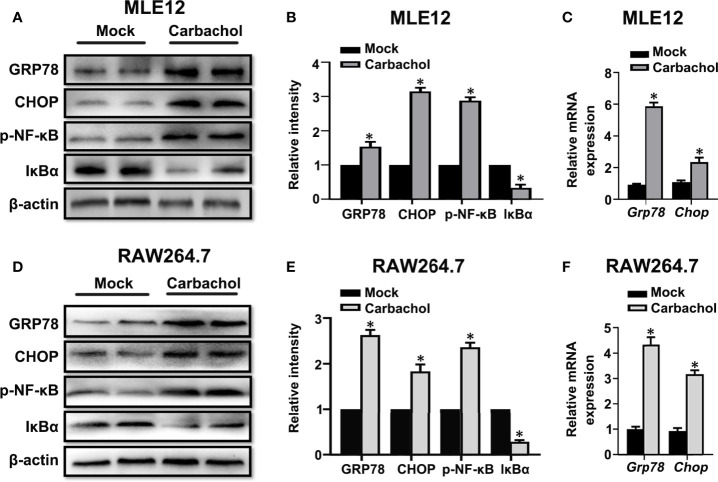
Carbachol stimulates ER stress in lung epithelial cells and macrophage. **(A, B)** Representative immunoblots of GRP78, CHOP, p-NF-κB p65 and IκBα in MLE12 cells and densitometric analyses of GRP78, CHOP, p-NF-κB p65 and IκBα. MLE12 cells were treated with 50 μM carbachol or non-treated for 24h. **(C)** Levels of GRP78, CHOP mRNA in MLE12 cells. **(D, E)** Representative immunoblots of GRP78, CHOP, p-NF-κB p65 and IκBα in RAW264.7 cells and densitometric analyses of GRP78, CHOP, p-NF-κB p65 and IκBα. RAW264.7 cells were treated with 50 μM carbachol or non-treated for 24h. **(F)** Levels of GRP78, CHOP mRNA in RAW264.7 cells. Data are expressed as means ± SD from 3 independent experiments. *P < 0.05 *vs*. Mock group.

To verify whether carbachol induce ER stress,we examined if carbachol affects changes in GRP78 and CHOP levels. Indeed, carbachol treatment produced robust upregulation of both GRP78 and CHOP protein and mRNA levels in both MLE12 (**Figures 10A–C**) and RAW264.7 cells (**Figures 10D–F**). Moreover, phosphorylation of NF-κB and downregulation of IκBα suggested that NF-κB signaling was also activated by carbachol (**Figures 10A, B, D, E**).

**Figure 11:**
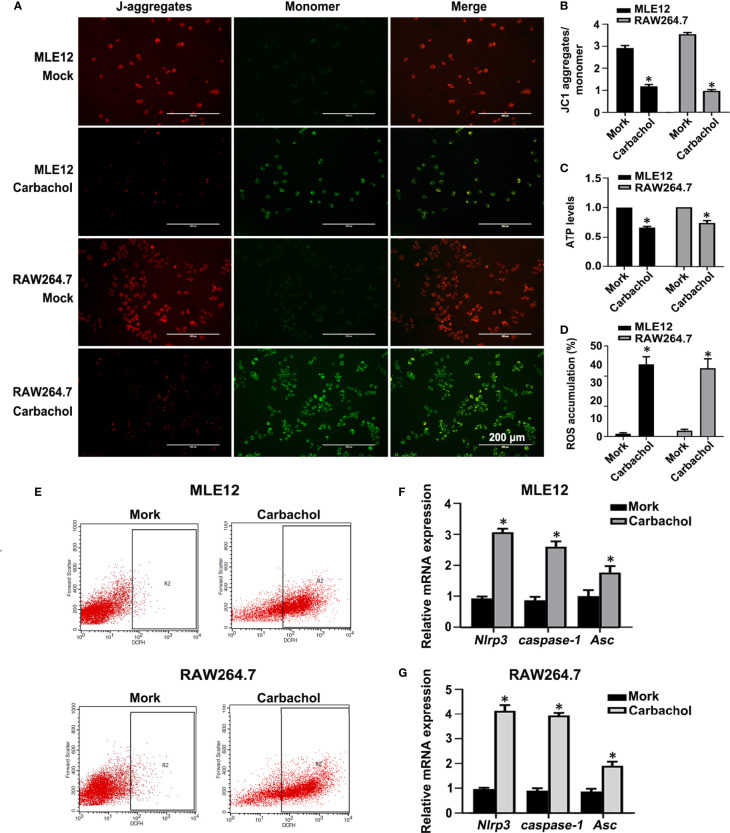
Carbachol stimulates mitochondrial dysfunction and NLRP3 inflammasome activation in lung epithelial cells and macrophage. **(A, B)** Fluorescence microscopy and the ratio of JC-1 staining (red, J-aggregates; green, monomer) in MLE12 and RAW264.7 cells after treating with 50 μM carbachol or non-treated for 24h, Scale bar: 200μm. **(C)** Levels of ATP. **(D, E)** Levels of ROS were determined by flow cytometry. **(F)** Levels of NLRP3, caspase-1and Asc mRNA in MLE12 cells. **(G)** Levels of NLRP3, caspase-1 and Asc mRNA in RAW264.7 cells. Data are expressed as means ± SD from 3 independent experiments. Mann-Whitney U test was used for caspase-1 mRNA comparison between two groups from MLE12 cells, because the data were non-normally distributed. *P < 0.05 *vs*. Mock group.

We also confirmed the effects of carbachol treatment on mitochondrial dysfunction in MLE12 and RAW264.7 cells. As anticipated, measuring the changes in Δψm, ATP levels and ROS production showed that cells treated with carbachol had decreased Δψm and ATP levels while ROS production was up-regulated (**Figures 11A–E**). Furthermore, indicative of NLRP3 inflammasome activation, exposing MLE12 and RAW264.7 cells to carbachol resulted in increased levels of NLRP3, caspase-1 and ASC mRNA (**Figures 11F, G**). Thus, the effects of carbachol on ER stress, mitochondrial dysfunction and NLRP3 inflammasome activation *in vitro* dovetail with the findings that IP3R mediates these same processes *in vivo*.

## Discussion

An exacerbated inflammatory response is viewed as the key process in the pathogenesis of VILI (11, 37). Nevertheless, how stretch-induced damage in alveolar cells leads to this inflammatory response has not been clearly elucidated. In the current study, we showed that IP3R/Ca2+ dysregulation occurs in a mouse model of VILI. Prominently, we found that restoring IP3R and Ca2+ homeostasis by pretreatment with 2-APB or BAPTA-AM could alleviate HTV-induced lung injury and inflammation. Moreover, 2-APB treatment in this model prevented ER stress and improved mitochondrial dysfunction caused by HTV. As further proof of concept, treatment of cultured epithelial cells and macrophage with carbachol to stimulate Ca2+ release from the ER, results in release of inflammatory mediators, with ER stress and mitochondrial dysfunction. This study has therefore revealed a novel inflammatory mechanism involved in the development of VILI together with suggesting potential strategies to prevent or treat VILI.

The ER is considered to be the most important intracellular Ca2+ store (38). The channels in the ER finely regulate the intracellular Ca2+ homeostasis (39, 40). Here IP3R, which is ubiquitously expressed non-selective cation channel, is responsible for the large conductance release of ER Ca2+ stores in all cell types (41, 42). However, the sustained release of Ca2+ through IP3R can be pathologic, causing cell damage and even inducing death (25, 43). Numerous studies suggest that IP3R/Ca2+ dysregulation represents an important inflammatory signal. For example, Jonathan and colleagues (44) showed that the activation of the CaSR/PLC/IP3R signaling pathway increases NOD1 inflammatory responses after bacterial infections. Furthermore, Wang et al. (45) found that TMEM16A overexpression increases IL-6 secretion *via* IP3R/Ca2+/NFκB signaling activation in pancreatic acinar cells. Studies such as these have led to considerable interest in the relationship between IP3R and VILI and as disclosed here, we found that HTV induced high IP3R1 expression in the mouse lungs. Moreover, the use of IP3R agonists and antagonists was enlightening, where pretreatment with either carbachol or 2-APB prior to ventilation significantly exacerbated or prevented lung injury, respectively. These findings clearly implicate IP3R in the development and maintenance of VILI.

Most studies have shown that IP3R is mainly expressed in the ER and MAM, and we also observed that after ventilation with HTV, IP3R1 increased in these two subcellular organelles. Thus, it is possible that HTV-induced IP3R pathological activity results from ER to cytoplasmic Ca2+ release, leading to cytoplasmic Ca2+ overload. On the other hand, IP3R is also required for MAM formation (15) which is also the site for IP3R-mediated transfer of Ca2+ to mitochondria. Excessive Ca2+ flux can also result in mitochondrial overload and as a consequence, both processes can conceivably lead to a decrease in [Ca2+]ER. Surprisingly, we also found that HTV enhances the formation of MAM in key lung cell types, which means HTV makes ER and mitochondria more closely related. These possibilities are not mutually exclusive, and indeed, we observed that Ca2+ levels in the cytoplasm and mitochondria increased dramatically in HTV mice, while [Ca2+]ER decreased. Again, the use of carbachol and 2-APB proved instructive, where carbachol pretreatment increased [Ca2+]ER efflux while by 2-APB prevented this process. Moreover, it has been well documented that Ca2+ homeostasis changes in the ER and mitochondria effect their respective functions, and our results propose this phenomenon occurs during VILI.

ER Ca2+ homeostasis is essential for maintaining adequate stores to ensure proper protein folding and trafficking. When the Ca2+ concentration in the ER is low, most ER chaperones which are Ca2+-dependent lose their activity, which results in the activation of ER stress/UPR (46). Previously, we reported that MV with HTV activates the IRE1α/TRAF2/NF-κB signaling pathway which was responsible for the release of high concentrations of inflammatory mediators, including IL-1β, IL-6 and TNF-α (8). However, it was unclear whether IP3R/Ca2+dysregulation triggers ER stress-induced inflammation and lung injury during VILI. In current study, HTV induced high expression of both IRE1α and PERK pathways-related proteins and these changes could be inhibited by pretreated with 2-APB. Furthermore, there was accompanying activation of NF-κB inflammatory pathway. The same paradigm was revealed *in vitro* using both lung epithelial and macrophage cells, where carbachol promoted ER stress, activation of NF-κB and stimulated the release of inflammatory factors. These data support that IP3R/Ca2+ dysregulation contributes to ER stress and activate NF-κB inflammatory pathway in lung cells.

High IP3R expression and increased MAM formation play pivotal roles in propagation of Ca2+ signals into the mitochondrial matrix. A transient increase in Ca2+ levels activates matrix enzymes and stimulates oxidative phosphorylation, but sustained exposure to high Ca2+ concentrations is often detrimental for mitochondrial function (31, 46). Alterations of mitochondrial function not only impact cellular metabolism but also play a role in the initiation of inflammatory signaling (47, 48). From the perspective of this study, mitochondrial ROS generation has been documented to activate the NLRP3 inflammasome, which is involved in inflammation and implicated in VILI (35, 36). However, whether IP3R/Ca2+ dysregulation was cause or effect in these processes was unclear. We established that HTV-treatment decreased Δψm and ATP levels while inducing NLRP3 inflammasome activation. Moreover, pretreating mice with 2-APB before HTV improved mitochondrial function and alleviated NLRP3 inflammasome activation, indicative of a causal role for IP3R/Ca2+ dysregulation. Furthermore, both mitochondrial dysfunction and NLRP3 inflammasome activation could be recapitulated *in vitro* by treating epithelial cells and macrophage with carbachol. Therefore, altered IP3R/Ca2+ fluxes may have a role in mitochondrial dysfunction and inflammation in lungs.

However, the mechanism of activation of IP3R in VILI remains unclear. In our previous research (49, 50), Toll-like Receptor 4 (TLR4), which is a member of the TLR family located on the membrane of cells. TLR4 activates when alveolar cells are overstretched. According to the literature (51, 52), TLR4-mediated signaling can lead to rapid activation of phosphoinositide 3-kinase (PI3K), subsequent phosphorylation and activation of Phospholipase C (PLC) (53). PLC activation has been shown to cause the production of IP3 (54), a Ca2+-releasing second messenger, which is essential for IP3R activation (55, 56). Thus, we hypothesize that such a robust increase of IP3R expression after HTV would be regulated by TLR4/PI3K/IP3 signaling pathway. In addition, it’s worth thinking about the “ Ca2+ re-entry” mechanisms. Studies have shown that the depletion of the ER Ca2+ stores stimulate store-operated Ca2+ entry (SOCE) (57–59), which is a major mechanism of Ca2+ import from extracellular to intracellular space to maintain calcium homeostasis in cells. In our case, however, whether this compensatory mechanism has been disrupted or weakened, we need further research.

### Limitations

There are several limitations that warrant further discussion. Firstly, vital signs of mice were assessed indirectly during ventilation in this study. Physiological parameters such as blood pressure and arterial blood gases are what we need to improve in the future research. Secondly, we did not set up the group that MV with low tidal volume (LTV). Although in our previous studies (8, 49), no histological differences and inflammation were noted between non-ventilated mice and LTV mice. But we still cannot exclude MV with LTV affects IP3R/Ca2+ dysregulation, then to make sure that these adverse reactions are completely due to overstretch. Thirdly, without seeing the effect of carbachol alone *in vivo*, we cannot ensure if carbachol has toxic effects on animals. Again, we did not set up the group with vehicle only, even though we used a very small dose (1%) of DMSO, which has been reported to cause cellular toxicity when concentrations >10% (v/v) (60). But we still cannot ensure if vehicle has side effects to animals in this study. Fourthly, although 2-APB blocks IP3R-activated Ca2+ release, it is likely that its effects result from the inhibition of TRP channels (61). Therefore, we need to examine other related Ca2+ channels more comprehensively. At last, studies using gene knockdown mice and mechanical stretch cells may offer further insights and allow to dissect if IP3R or other potential related effects have dominated VILI development and maintainment (61).

## Conclusion

**Figure 12:**
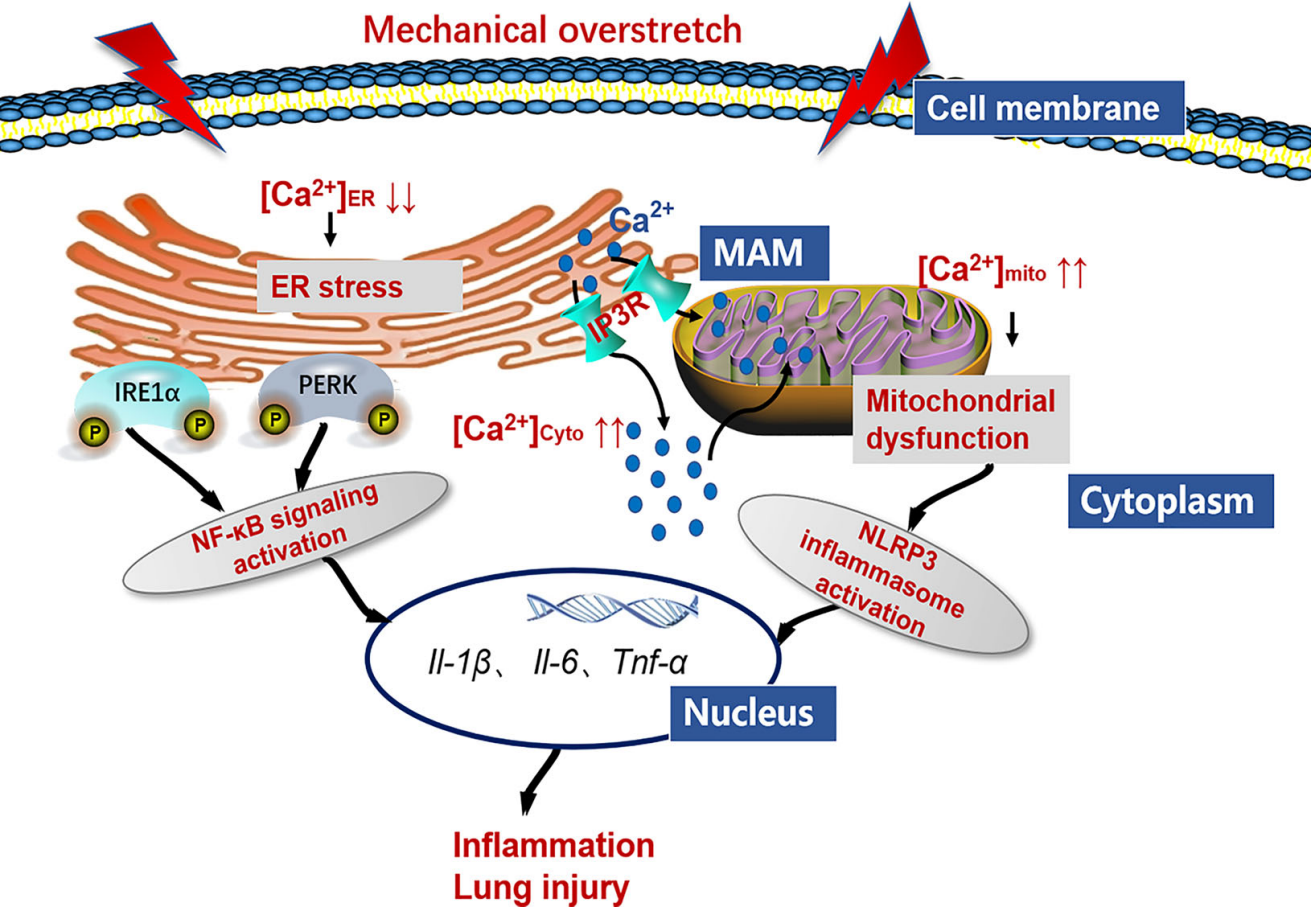
Working model detailing the role of IP3R/Ca2+ in VILI. Mechanical overstretch induces cell inflammation through an accelerated ER-to-cytosolic and ER-to-mitochondria efflux of Ca2+ through IP3R, followed by ER stress and mitochondrial dysfunction.

In conclusion, we demonstrate that HTV-induced mechanical overstretch activates IP3R1, and IP3R-linked Ca2+ dysregulation contributes to ER stress and mitochondrial dysfunction. On the one hand, ER stress contributes to IRE1α and PERK signaling activation, which increases the releases of NF-κB-related inflammatory factors and aggravates lung injury. On the other hand, mitochondrial dysfunction leads to NLRP3 inflammasome activation, which are involved in the VILI pathogenesis (**Figure 12**). We also establish the concept that antagonizing IP3R offers a means to prevent or treat VILI.

## Data Availability Statement

The raw data supporting the conclusions of this article will be made available by the authors, without undue reservation.

## Ethics Statement

The animal study was reviewed and approved by Institutional Animal Care and Use Committee of Guangxi Medical University Cancer Hospital.

## Author Contributions

LP designed the overall study. LY and QZ performed the experiments and drafted the manuscript. ML, RM, HC, FL, and ZL performed the data analysis and contributed reagents and materials. All authors contributed to the article and approved the submitted version.

## Funding

This work was supported by the National Natural Science Foundation of China (81970078), Guangxi Natural Science Foundation of China (2020GXNSFBA297058), and the International Communication of Guangxi Medical University Graduate Education (2019).

## Conflict of Interest

The authors declare that the research was conducted in the absence of any commercial or financial relationships that could be construed as a potential conflict of interest.

## Publisher’s Note

All claims expressed in this article are solely those of the authors and do not necessarily represent those of their affiliated organizations, or those of the publisher, the editors and the reviewers. Any product that may be evaluated in this article, or claim that may be made by its manufacturer, is not guaranteed or endorsed by the publisher.

## Supplementary Material

Supplementary Figure 1 Assessment of the dose-dependent effects of BAPTA-AM on cytoplasmic Ca2+ levels. **(A, B)** Fluo-4 AM labeling and quantification analysis in group CON and HTV mice and HTV-treated mice administered with BAPTA-AM with 1.25, 2.5 and 5 mg/kg respectively. Data are expressed as means ± SD (n = 6 per group). **P* < 0.05 *vs*. CON group. ^#^ *P* < 0.05 *vs*. HTV group.

Supplementary Figure 2 Evaluation of the cytotoxic effects of carbachol on MLE12 and RAW264.7 cells. **(A)** Cell viability of MLE12 and RAW264.7 cells after treating with different doses of carbachol as observed by CCK8 assays. **(B)** Morphological changes and reduced cell populations of MLE12 and RAW264.7 cells treated with different concentrations of carbachol. Scale bar: 200 μm. **(C)** Detection of apoptotic MLE12 and RAW264.7 cells after 50 μM carbachol by flow cytometry based-annexin V- FITC/PI analysis. Data are expressed as means ± SD from 3 independent experiments. **P* < 0.05 *vs*. Mock group.

## Abbreviations

MV, mechanical ventilation; ARDS, acute respiratory distress syndrome; HTV, high tidal volume; VILI, ventilator-induced lung injury; ER, endoplasmic reticulum; UPR, unfolded protein response; ATP, adenosine triphosphate; ROS, reactive oxygen species; 2-APB, 2-aminoethoxydiphenyl borate; Ca2+, calcium; [Ca2+]cyto, cytoplasmic Ca2+ concentration; [Ca2+]mito, mitochondria Ca2+ concentration; [Ca2+]ER, endoplasmic reticulum Ca2+ concentration; IP3R, Inositol 1,4,5-trisphosphate receptor; MAMs, mitochondrial-associated-membranes; BALF, bronchoalveolar lavage fluid; H&E, Hematoxylin-Eosin; W/D, wet/dry; TEM, transmission electron microscopy; BCA, bicinchonininc acid; IL-1β, interleukin-1 beta; IL-6, interleukin-6; TNF-α, tumor necrosis factor-alpha; DMEM, Dulbecco’s modified Eagle’s medium; V-FITC/PI, V-fluorescein isothiocyanate/propidium iodide; Δψm, mitochondrial membrane potential; PVDF, polyvinylidene fluoride; ECL, enhanced chemiluminescence; SD, standard deviation.
