# Supplementary figures and images for: Inhibition of IP3R/Ca2+ Dysregulation Protects Mice From Ventilator-Induced Lung Injury via Endoplasmic Reticulum and Mitochondrial Pathways
Source: Front Immunol. 2021 Sep 15;12:729094. doi: 10.3389/fimmu.2021.729094 (PMC8479188; doi:10.3389/fimmu.2021.729094)

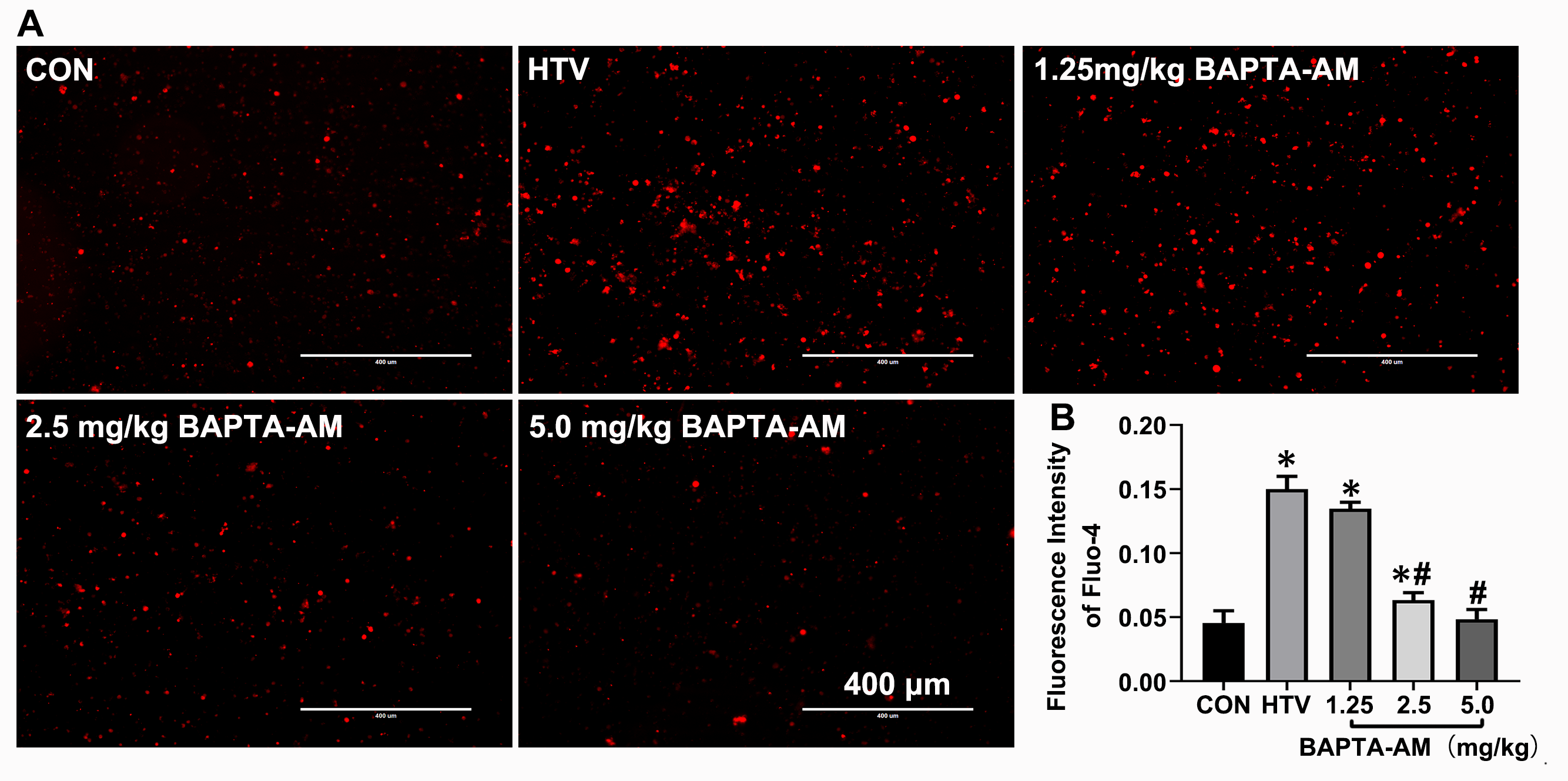

Supplement: Supplementary Figure 1 — Assessment of the dose-dependent effects of BAPTA-AM on cytoplasmic Ca2+ levels. (A, B) Fluo-4 AM labeling and quantification analysis in group CON and HTV mice and HTV-treated mice administered with BAPTA-AM with 1.25, 2.5 and 5 mg/kg respectively. Data are expressed as means ± SD (n = 6 per group). *P < 0.05 vs. CON group. # P < 0.05 vs. HTV group. [file Image_1.tif]

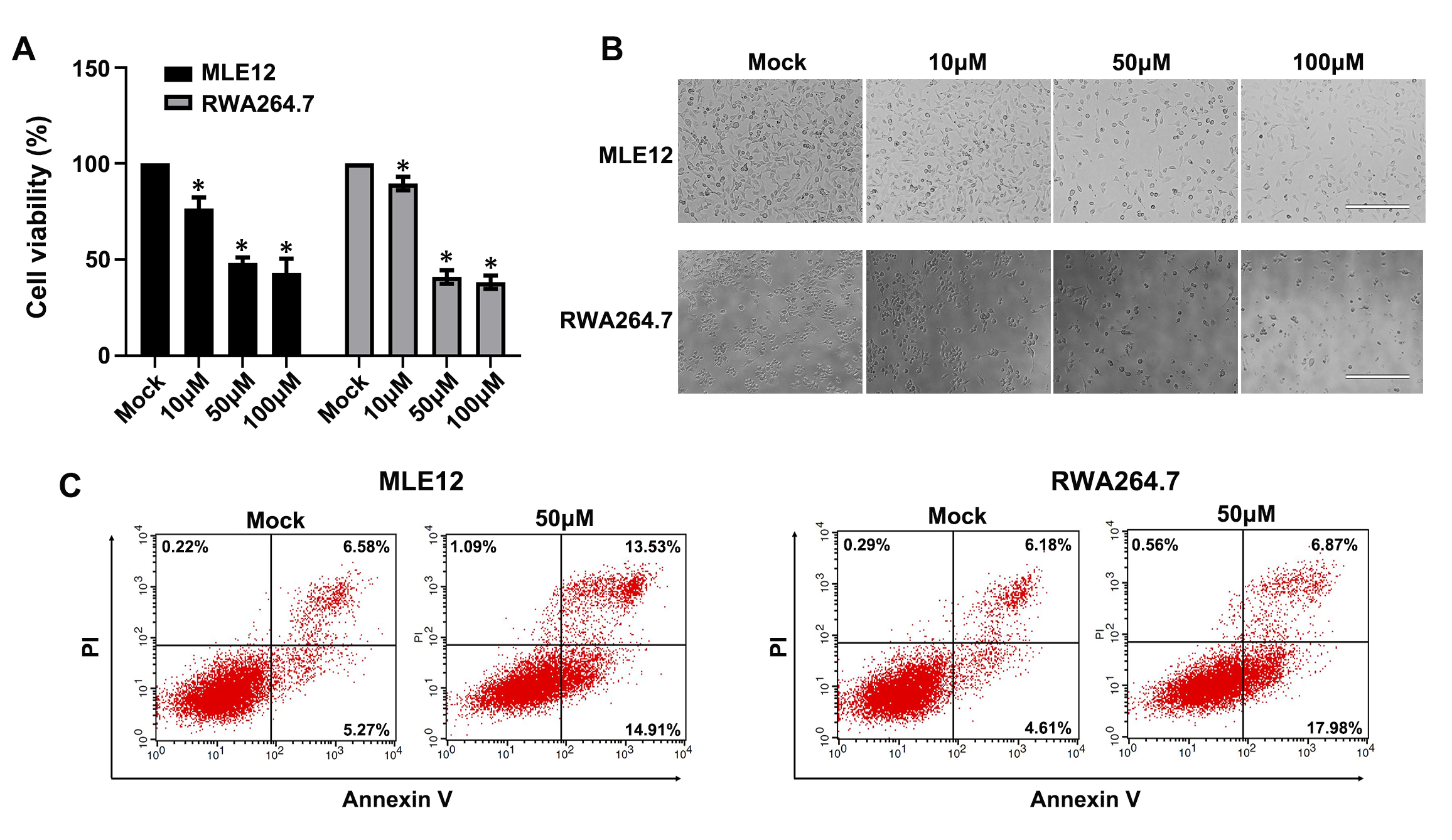

Supplement: Supplementary Figure 2 — Evaluation of the cytotoxic effects of carbachol on MLE12 and RAW264.7 cells. (A) Cell viability of MLE12 and RAW264.7 cells after treating with different doses of carbachol as observed by CCK8 assays. (B) Morphological changes and reduced cell populations of MLE12 and RAW264.7 cells treated with different concentrations of carbachol. Scale bar: 200 μm. (C) Detection of apoptotic MLE12 and RAW264.7 cells after 50 μM carbachol by flow cytometry based-annexin V- FITC/PI analysis. Data are expressed as means ± SD from 3 independent experiments. *P < 0.05 vs. Mock group. [file Image_2.tif]
